# Chloramphenicol enhances Photosystem II photodamage in intact cells of the cyanobacterium *Synechocystis* PCC 6803

**DOI:** 10.1007/s11120-020-00784-1

**Published:** 2020-09-26

**Authors:** Sandeesha Kodru, Ateeq ur Rehman, Imre Vass

**Affiliations:** 1 Institute of Plant Biology, Biological Research Centre, Temesvari krt. 62, Szeged, 6726 Hungary; 2 Doctoral School of Biology, University of Szeged, Szeged, Hungary

**Keywords:** Chloramphenicol, Photoinhibition, Photosystem I, Photosystem II, Superoxide, Synechocystis PCC 6803

## Abstract

The effect of chloramphenicol, an often used protein synthesis inhibitor, in photosynthetic systems was studied on the rate of Photosystem II (PSII) photodamage in the cyanobacterium *Synechocystis* PCC 6803. Light-induced loss of PSII activity was compared in the presence of chloramphenicol and another protein synthesis inhibitor, lincomycin, by measuring the rate of oxygen evolution in *Synechocystis* 6803 cells. Our data show that the rate of PSII photodamage was significantly enhanced by chloramphenicol, at the usually applied 200 μg mL^−1^ concentration, relative to that obtained in the presence of lincomycin. Chloramphenicol-induced enhancement of photodamage has been observed earlier in isolated PSII membrane particles, and has been assigned to the damaging effect of chloramphenicol-mediated superoxide production (Rehman et al. 2016, Front Plant Sci 7:479). This effect points to the involvement of superoxide as damaging agent in the presence of chloramphenicol also in *Synechocystis* cells. The chloramphenicol-induced enhancement of photodamage was observed not only in wild-type *Synechocystis* 6803, which contains both Photosystem I (PSI) and PSII, but also in a PSI-less mutant which contains only PSII. Importantly, the rate of PSII photodamage was also enhanced by the absence of PSI when compared to that in the wild-type strain under all conditions studied here, i.e., without addition and in the presence of protein synthesis inhibitors. We conclude that chloramphenicol enhances photodamage mostly by its interaction with PSII, leading probably to superoxide production. The presence of PSI is also an important regulatory factor of PSII photodamage most likely via decreasing excitation pressure on PSII.

**Electronic supplementary material:**

The online version of this article (10.1007/s11120-020-00784-1) contains supplementary material, which is available to authorized users.

## Introduction

Photosynthesis is a process in which green plants, algae, and cyanobacteria utilize energy from sunlight to produce carbohydrates from carbon dioxide and water. This process is the ultimate source of energy for all plants to drive their metabolic processes. Too much light reaching the photosynthetic apparatus can cause photodamage and finally can lead to the death of a cell, a phenomenon called photoinhibition (Arntzen et al. 1984; Aro et al. 1993; Vass and Aro 2008; Zavafer et al. 2017). A major impact on the photosynthetic machinery under high light conditions is the impairment of electron transport in the Photosystem II (PSII) complex, as well as damage of its D1 reaction center subunit (Ohad et al. 1984; Prasil et al. 1992; Aro et al. 1993). Important mediators of photodamage in plant cells are the various reactive oxygen species (ROS), such as singlet excited oxygen, free radicals (superoxide and hydroxyl ions), superoxide and peroxides, which are produced mainly in the chloroplasts and mitochondria (Apel and Hirt 2004; Krieger-Liszkay 2005; Krieger-Liszkay et al. 2008; Pospísil 2012; Vass 2012). The activity of the photodamaged PSII complex can be restored via the so-called PSII repair cycle, in which de novo synthesis of the D1 subunits plays a key role (Aro et al. 1993; Baena-Gonzalez and Aro 2002; Komenda et al. 2007; Nixon et al. 2010; Vass 2012; Järvi et al. 2015; Li et al. 2018).

Light stress to PSII becomes a problem for photosynthetic activity when the rate of photodamage exceeds the rate of repair processes. Therefore, it is important to monitor separately the rates of photodamage and of the protein synthesis-dependent repair. Decoupling of photodamage and repair can be achieved by protein synthesis inhibitors, such as lincomycin or chloramphenicol, which block the initiation of protein synthesis and of peptide bond formation, respectively (Contreras and Vazquez 1977). These protein synthesis inhibitors can be applied both in chloroplasts (Mulo et al. 2003; Chow et al. 2005; Tikkanen et al. 2014) and cyanobacterial cells (Constant et al. 1997; Nishiyama et al. 2001, 2005; Takahashi and Murata 2005; Takahashi et al. 2009; Sicora et al. 2003). While there are no reports concerning the participation of lincomycin in photosynthetic electron transport, chloramphenicol has been reported to accept electrons from the acceptor side of Photosystem I (PSI) and to transfer them to molecular oxygen leading to superoxide production (Okada et al. 1991). Although O_2_^**·**−^ has lower reactivity than other ROS, it can induce damage to proteins and membrane components due to its ability to produce highly oxidizing radicals (Pospisil et al. 2019). This side effect of chloramphenicol has been considered as a source of a potential artifact by several research groups, who used lincomycin instead of chloramphenicol in photoinhibition studies (Constant et al. 1997; Tyystjärvi and Aro 1996; Tyystjarvi et al. 2002; Chow et al. 2005; Miyata et al. 2012; Campbell and Tyystjärvi 2012; Tikkanen et al. 2014). However, other groups kept using chloramphenicol in measurements of PSII photodamage, and often obtained results which were contradictory to those studies in which lincomycin was used (Nishiyama et al. 2001, 2005; Takahashi and Murata 2005; Takahashi et al. 2009). It is also of note that while chloramphenicol was used at 30–65 or 100 μg mL^−1^ in some early investigations, it was applied at 200 μg mL^−1^ or higher concentrations in the majority of photosynthesis related studies.

Recently, we have shown in isolated thylakoid membrane systems that chloramphenicol can accept electrons not only from PSI but also from the acceptor side of PSII, and deliver them to molecular oxygen leading to superoxide production (Rehman et al. 2016). In addition, the presence of 200 μg mL^−1^ chloramphenicol enhances photodamage of PSII electron transport in the isolated systems, an effect that is reversible by superoxide dismutase (Rehman et al. 2016), pointing to the damaging role of superoxide in the process of photoinhibition.

In the present work, we have extended our earlier studies from isolated thylakoid membrane preparations to intact cell cultures of the cyanobacterium *Synechocystis* PCC 6803, as well as to its PSI-less mutant. Our data show that the presence of 200 μg mL^−1^ chloramphenicol enhances photodamage of PSII in intact *Synechocystis* cells even in the absence of PSI. As a consequence, chloramphenicol, when applied at 200 μg mL^−1^ or higher concentrations, can lead to artifacts in studies aiming at the determination of the true rate of PSII photodamage in intact systems.

## Materials and methods

### Cell cultures

Wild-type *Synechocystis* sp. PCC 6803 (which will be referred to as WT *Synechocystis*) cells were propagated in BG-11 growth medium in a rotary shaker at 30 °C under a 3% CO_2_-enriched atmosphere. The intensity of white light during growth was 40 µmol photons m^−2^ s^−1^. Cells in the exponential growth phase (A_580_ of 0.8–1) were used. The PSI-less strain of *Synechocystis*, was produced by Wim Vermaas similarly to that described earlier (Shen et al. 1993), but using spectinomycin instead of chloramphenicol as selective antibiotic. PSI-less cells were grown at low light intensity (of 5 µmol photons m^−2^ s^−1^) in the presence of 5 mM glucose and 25 μg mL^−1^ spectinomycin. As a control for the PSI-less strain, WT cells were also cultured under the same conditions, i.e., 5 µmol photons m^−2^ s^−1^ light intensity in the presence of 5 mM glucose when indicated.

### Light treatment

Cells were harvested by centrifugation at 8000×*g* for 10 min and resuspended in 100 mL fresh BG-11 medium at 5 μg Chl mL^−1^ concentration. Before starting high light treatment, cells were incubated for 1 h under growth light (40 μmol photons m^−2^ s^−1^) followed by measuring the control value of oxygen evolution, which was used as zero time point for the high light treatment. In experiments with protein synthesis inhibitors, cells were incubated at growth light for 40 min in the absence of inhibitor followed by an additional 20 min incubation in the presence of the inhibitor before measuring the control value of oxygen evolution rate. For photoinhibitory treatment, cells were illuminated with 500 μmol photons m^−2^ s^−1^ white light without additions, in the presence of the protein synthesis inhibitor lincomycin (300, or 400 μg mL^−1^) or chloramphenicol (200 µg mL^−1^). The temperature during illumination was maintained at 30 °C.

### Oxygen evolution rate measurements

Steady-state O_2_ evolution rates were measured by using a Hansatech DW2 O_2_ electrode at 30 °C under illumination with 2300 μmol m^−2^ s^−1^ light intensity in the presence of 0.5 mM DMBQ as an artificial electron acceptor.

### Statistical analysis

Statistical analysis was performed by one way ANOVA using the Turkey test for means comparison. The calculations were done by the Origin 2018 graphics software.

## Results

### Chloramphenicol enhances PSII photodamage in comparison to lincomycin in a concentration dependent way in WT *Synechocystis*

**Fig. 1:**
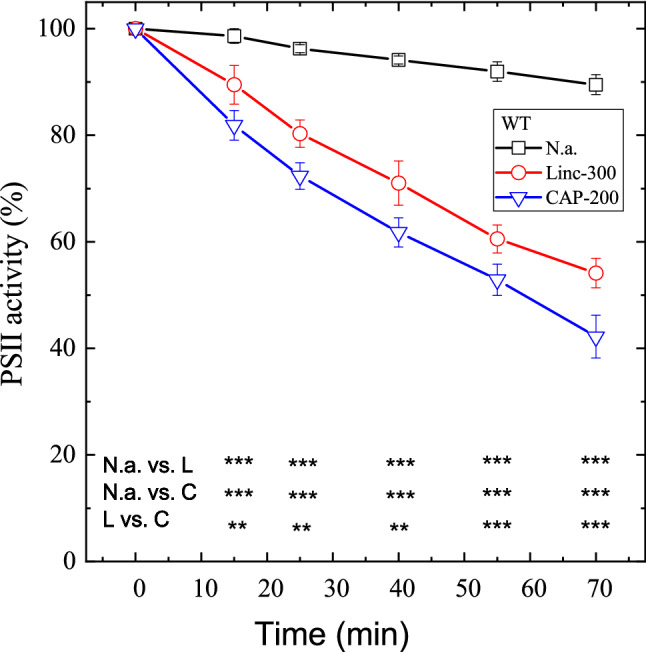
Differential effects of lincomycin and chloramphenicol on photoinhibitory activity loss in WT *Synechocystis* cells. WT *Synechocystis* cultures were exposed to 500 μmole photons m^−2^ s^−1^ intensity illumination. The cultures were left untreated (squares), treated with 300 μg mL^−1^ lincomycin (circles), or 200 μg mL^−1^ chloramphenicol (down triangles). PSII activity was assessed by measuring the rate of oxygen evolution in the presence of 0.5 mM DMBQ as an artificial acceptor. Data are shown as a percentage of the initial PSII activity, which was obtained from the growth-light-adapted cultures before the onset of high light illumination. The asterisk signs indicate the time points where the PSII activity values are significantly (*p < 0.05, **p < 0.01, ***p < 0.001) different between the untreated (n.a.), lincomycin (L) or chloramphenicol (C)-treated samples

In order to quantify the effect of 200 μg mL^−1^ chloramphenicol on the rate of PSII photodamage, WT *Synechocystis* cells were exposed to 500 μmol photons m^−2^ s^−1^ illumination without addition, and in the presence of either lincomycin or chloramphenicol as protein synthesis inhibitor. High light alone induced only a small extent of PSII activity loss, as quantified by the rate of oxygen evolution in the presence of 0.5 mM DMBQ as an artificial acceptor. After 70 min illumination, PSII activity declined to 90% of its initial value (Fig. 1, squares). In the presence of lincomycin, PSII inactivation was enhanced and the residual activity declined to 55% of its initial value after 70 min (Fig. 1, circles). When 200 μg mL^−1^ chloramphenicol was used as a protein synthesis inhibitor instead of lincomycin, the extent of PSII deactivation was further enhanced, and the residual activity after 70 min illumination was only ca. 40% of its initial value (Fig. 1, down triangles). The lower part of Fig. 1 shows that the differences in PSII activity values were statistically significant at each time point for the no addition vs. lincomycin, no addition vs. chloramphenicol, and lincomycin vs. chloramphenicol treatments at *p* < 0.01 (or 0.001) significance level (see also Supplementary Table 1).

In order to make sure that the enhanced loss of PSII activity in the presence of chloramphenicol was a real effect and not caused by partial inhibition of protein synthesis in the presence of the applied 300 μg mL^−1^ concentration of lincomycin, the photoinhibitory experiments were also performed in the presence of 400 μg mL^−1^ lincomycin. These data (Supplementary Fig. 1) confirmed that lincomycin at 300 μg mL^−1^ concentration was sufficient for full inhibition of protein synthesis. Therefore, the enhancement of photodamage in the presence of 200 μg mL^−1^ chloramphenicol is caused by an effect, which is unrelated to protein synthesis inhibition.

In the majority of photosynthetic applications chloramphenicol is applied in 200 μg mL^−1^ or higher concentrations. However, in some studies 100 μg mL^−1^ or even as low as 30–50 μg mL^−1^ was also used (Supplementary Table 1). Therefore, we aimed to check if these lower concentrations also enhance PSII photodamage. Our data show that PSII activity loss in the presence of 100 μg mL^−1^ chloramphenicol was practically identical with that observed in the presence of 300 μg mL^−1^ lincomycin (Supplementary Fig. 1). In contrast, 50 μg mL^−1^ chloramphenicol resulted in a slower loss of PSII activity than lincomycin. In addition, some recovery was also observed under low light conditions following the photoinhibitory treatment, which indicates that 50 μg mL^−1^ chloramphenicol is not sufficient for complete blocking of PSII repair under our experimental conditions.

The rates of PSII activity loss were calculated by fitting the activity curves with a single exponential decay function (*A*_*i*_*e*^−kt^, where *A*_*i*_ is the initial activity, *k* is the rate of activity loss, and *t* is time). The calculated rates are summarized in Table 1, which show that the *k* = 0.0016 min^−1^ rate of photodamage, which was observed in the absence protein synthesis inhibitors was increased to 0.0090 min^−1^ in the presence of 300 μg mL^−1^ lincomycin. Practically the same photodamage rate (0.0083 min^−1^) was obtained in the presence of 100 μg mL^−1^ chloramphenicol. However, the damage rate was increased by ca. 50% (to 0.0120 min^−1^) when 200 μg mL^−1^ chloramphenicol was applied showing a concentration dependence of chloramphenicol-induced photodamage.

**Table 1:** Rates of PSII photodamage

Sample	Rate of photodamage (min^−1^)	
	Mean	Stdev
WT	0.0016	1.42E−04
WT+300 linc	0.0088	3.13E−04
WT+400 linc	0.0090	9.77E−04
WT+100 CAP	0.0083	1.24E−03
WT+200 CAP	0.0120	6.59E−04
WT-gluc	0.0015	1.36E−04
WT-gluc+300 linc	0.0085	3.65E−04
WT-gluc+200 CAP	0.0119	8.23E−04
PSI-less	0.0035	5.41E−04
PSI-less+300 linc	0.0152	5.51E−04
PSI-less+200 CAP	0.0217	1.23E−03

*Synechocystis* cultures were exposed to 500 μmole m^−2^ s^−1^ light and their PSII activity was followed as a function of illumination time by measuring the rate of oxygen evolution in the presence of DMBQ as artificial acceptor as shown in Fig. 1. PSII photodamage rates were calculated by fitting the PSII activity curves with a single exponential decay function (*A*_*i*_*e*^−*kt*^, where *A*_*i*_ is the PSII initial activity, *k* is the photodamage rate, and *t* is time) using the built in option of the Origin 2018 Pro software. The Stdev column shows the standard error of rate constants as calculated by the fitting software. The experiments were performed in photoautotrophically grown WT cells, without addition, and in the presence of protein synthesis inhibitors (300 and 400 μg mL^−1^ lincomycin, 200 μg mL^−1^ chloramphenicol, abbreviated as CAP). The photoinhibitory treatments were also performed in a PSI-less *Synechocystis* mutant line, which cannot grow photoautotrophically, and therefore was cultured photomixotrophically in the presence of 5 mM glucose (PSI-less-gl). As a control for the PSI-less mutant, the WT cells were also grown photomixotrophically (WT-gl). The PSI-less-gl and WT-gl cultures were exposed to the same high light and inhibitor treatments as the photoautotrophically grown WT

### Chloramphenicol enhances PSII photodamage in comparison to lincomycin in PSI-less *Synechocystis*

**Fig. 2:**
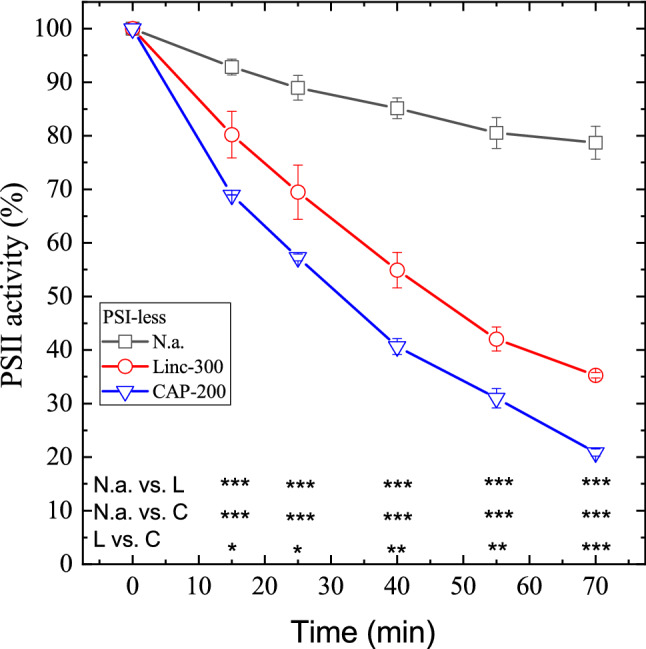
Differential effects of lincomycin and chloramphenicol on photoinhibitory activity loss in PSI-less *Synechocystis* cells. PSI-less *Synechocystis* cultures were exposed to 500 μmole photons m^−2^ s^−1^ intensity illumination. The cultures were left untreated (squares), treated with 300 μg mL^−1^ lincomycin (circles), or 200 μg mL^−1^ chloramphenicol (down triangles). PSII activity was assessed by measuring the rate of oxygen evolution in the presence of 0.5 mM DMBQ as an artificial acceptor. Data are shown as a percentage of the initial PSII activity, which was obtained from the growth-light-adapted cultures before the onset of high light illumination. The asterisk signs indicate the time points where the PSII activity values are significantly (*p < 0.05, **p < 0.01, ***p < 0.001) different between the untreated (n.a.), lincomycin (L) or chloramphenicol (C)-treated samples

In order to clarify if the photodamage-enhancing effect of chloramphenicol is related to its interaction with PSI or PSII, the light treatment experiments were also performed in a *Synechocystis* mutant, which lacks PSI. In contrast to the WT strain, high light exposure alone induced a substantial loss of PSII activity in the PSI-less mutant, which resulted in a decline to ca. 77% of the initial activity after a 70-min illumination (Fig. 2, squares). The addition of lincomycin enhanced the activity loss to ca. 35% of the initial activity after a 70-min illumination (Fig. 2, circles). In the presence of 200 μg mL^−1^ chloramphenicol the extent of PSII deactivation was further enhanced, leading to a residual activity of ca. 20% of the initial activity after 70 min (Fig. 2, down triangles). The lower part in Fig. 2 shows that the differences in PSII activity values were statistically significant in each time point for the no addition vs. lincomycin, no addition vs. chloramphenicol, and lincomycin vs. chloramphenicol treatments at *p* < 0.05 (0.01, or 0.001) significance level (see also Supplementary Table 2).

The calculated rates of PSII photodamage confirm that 200 μg mL^−1^ chloramphenicol enhances photodamage also in the PSI-less mutant (*k* = 0.0217 min^−1^) relative to that observed in the presence of lincomycin (*k* = 0.0152 min^−1^) (Table 1).

### The lack of PSI enhances PSII photodamage

Data in Figs. 1, 2 and Table 1 show that the light-induced loss of PSII activity is apparently enhanced in the PSI-less mutant when compared to the WT. However, we have to note that the growth conditions for the WT and the PSI-less strains were different (photoautotrophic growth at 40 μmol photons m^−2^ s^−1^ for the WT, and photomixotrophic growth in the presence of 5 mM glucose at 4 μmol photons m^−2^ s^−1^ for the PSI-less mutant). In order to clarify if the enhanced photodamage of PSII activity is due to the lack of PSI, or occurs as a consequence of different growth conditions, the light treatments were also performed in WT cells that were grown under the same conditions as the PSI-less mutant.

**Fig. 3:**
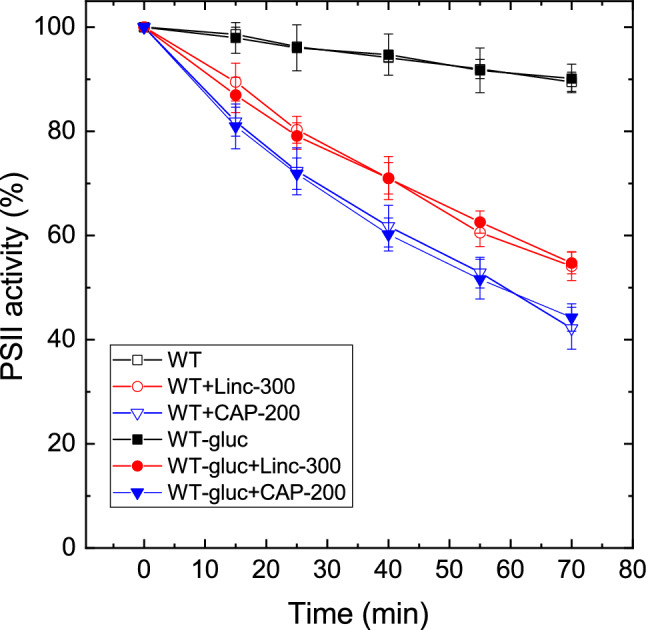
Effect of photoheterotrophic growth on photoinhibitory activity loss in WT *Synechocystis* cells. WT *Synechocystis* cultures were grown either in glucose-free BG-11 medium at 40 μmole photons m^−2^ s^−1^ light intensity (open symbols), or in the presence of 5 mM glucose at 5 µmole photons m^−2^ s^−1^ light intensity (closed symbols). The cultures were left untreated (squares), treated with 300 μg mL^−1^ lincomycin (circles) or 200 μg mL^−1^ chloramphenicol (down triangles) and were exposed to 500 μmole photons m^−2^ s^−1^ intensity illumination. PSII activity was assessed by measuring the rate of oxygen evolution in the presence of 0.5 mM DMBQ as artificial acceptor. Data are shown as percentage of the initial PSII activity, which was obtained from the growth-light-adapted cultures before the onset of high light illumination

**Fig. 4:**
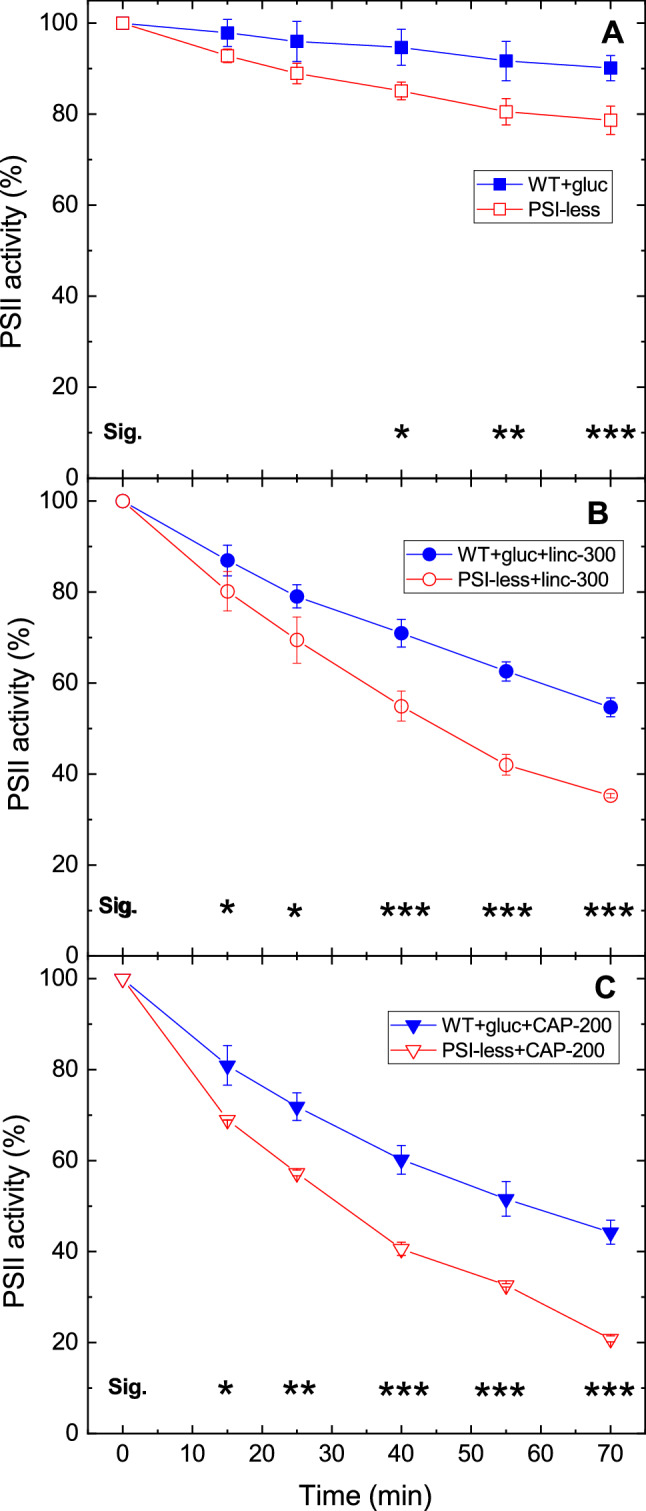
Differential light sensitivity of WT and PSI-less Synechocystis cells. WT (closed symbols) and PSI-less *Synechocystis* cultures (open symbols) were grown in the presence of 5 mM glucose at 5 µmole photons m^−2^ s^−1^ light intensity. The cultures were left untreated (squares), treated with 300 μg mL^−1^ lincomycin (circles), or 200 μg mL^−1^ chloramphenicol (down triangles) and were exposed to 500 μmole photons m^−2^ s^−1^ intensity illumination. PSII activity was assessed by measuring the rate of oxygen evolution in the presence of 0.5 mM DMBQ as artificial acceptor. Data are shown as percentage of the initial PSII activity, which was obtained from the growth-light-adapted cultures before the onset of high light illumination. The asterisk signs indicate the time points where the PSII activity values are significantly (*p < 0.05, **p < 0.01, ***p < 0.001) different between the WT and PSI-less samples

Growing the WT cells in the presence of glucose and low light did not affect their light sensitivity when compared to those grown photoautotrophically at standard light intensity (Fig. 3, see also Table 1). However, pairwise comparison of the activity loss curves in cells which were cultured under the same conditions (photomixotrophy and low light) reveal that PSII photodamage is significantly larger in the PSI-less strain than in the WT. This effect was observed in all three treatment conditions, i.e., without addition (Fig. 4a), in the presence of lincomycin (Fig. 4b) or chloramphenicol (Fig. 4c). The calculated rates of PSII photodamage are also ca. twofold higher in the PSI-less mutant than in the WT grown under photomixotrophic (or photoautotrophic) conditions (Table 1).

## Discussion

### Chloramphenicol enhances PSII photodamage

Our present data confirm that the rate of photodamage of PSII in intact *Synechocystis* cells is higher in the presence of the usually applied 200 μg mL^−1^ chloramphenicol than in the presence of lincomycin (Figs. 1, 2, 3). The data presented in Supplementary Fig. 1 exclude the possibility that this phenomenon would be caused by a sub-saturating concentration of lincomycin. However, the different action mechanisms of protein synthesis inhibition by lincomycin and chloramphenicol should also be considered for the explanation of the results. It is known that high light exposure results in elevated association of *psbA* mRNA with polysomes (Tyystjarvi et al. 2001) and also that lincomycin does not interact with ribosomes with bound nascent peptides (Contreras and Vazquez 1977) and therefore will not act on pre-formed polysomes. However, chloramphenicol is effective against peptide bond formation in ribosomes and polysomes (Contreras and Vazquez 1977). As a consequence, D1 copies which are pre-formed before the addition of lincomycin can be incorporated into the thylakoid membrane and can support PSII repair to some extent in spite of the presence of the protein synthesis inhibitor, whereas in the presence of chloramphenicol such an effect cannot occur. An interesting example of this phenomenon was the observation that after transferring high-light-exposed *Synechocystis* cells to low light conditions, the oxygen-evolving activity increased above the control level which persisted before the high light treatment, and this effect was not inhibited by lincomycin, only by chloramphenicol (Bentley et al. 2008). Based on the above-described difference in the action mechanisms of the two protein synthesis inhibitors, the smaller extent of PSII activity loss in the presence of lincomycin could in principle arise from a partial blocking of PSII repair due to the incorporation of pre-formed D1 copies, which were produced before the start of high light illumination, into the thylakoid membranes. However, under our experimental protocol cells were incubated in the presence of lincomycin (and chloramphenicol) for 20 min under standard growth light intensity (40 µmol photons m^−2^ s^−1^) before the onset of the high light treatment, which should be sufficient to empty the pre-D1 pool by the time strong illumination started. In addition, the effect of pre-D1 accumulation at growth light (40 µmol photons m^−2^ s^−1^) before lincomycin additions should be negligible for the repair of PSII under high light (500 µmol photons m^−2^ s^−1^) exposure. Therefore, we can safely conclude that lincomycin fully blocked PSII repair and the greater loss of PSII activity in the presence of 200 μg mL^−1^ chloramphenicol is related to a side effect of this protein synthesis inhibitor.

The side effect of chloramphenicol, which is concentration dependent, has been previously demonstrated in isolated PSII membranes (Rehman et al. 2016). Since this effect was reversible by SOD, which eliminated O_2_^**·**−^, it was concluded that chloramphenicol-mediated O_2_^**·**−^ production is the cause of chloramphenicol-enhanced photodamage in isolated systems (Rehman et al. 2016). SOD is a large enzyme (32.5 kDa molecular mass), which cannot penetrate through the cell wall of *Synechocystis*; therefore, SOD addition cannot be used to verify the involvement of O_2_^**·**−^ in chloramphenicol-induced damage in intact cells. On the other hand, based on the analogy with the results obtained in isolated PSII membrane particles, it is highly likely that O_2_^**·**−^ production in the presence of chloramphenicol is the main cause of enhanced photodamage also in the intact cells.

O_2_^**·**−^ has lower reactivity than other ROS (Pospisil et al. 2019); however, it can induce damage to proteins and membrane components due to its ability to produce highly oxidizing radicals. During spontaneous dismutation of O_2_^**·**−^ it forms H_2_O_2_, which in turn can produce the highly oxidizing hydroxyl radical (HO^**·**^). In addition, protonation of the anionic form of O_2_^**·**−^ leads to the formation of the perhydroxyl radical (HO_2_^**·**^), which is considered to be more reactive than O_2_^**·**−^ and damages polyunsaturated fatty acids, amino acids and nucleic acids by H_2_ abstraction (Aikens and Dix 1991).

O_2_^**·**−^ formation has been suggested to occur in PSII during high light exposure in the absence of added electron transport mediators (Pospísil 2012; Kale et al. 2017). In addition, O_2_^**·**−^-induced or O_2_^**·**−^-mediated damage of PSII core proteins (D1 and D2) has been observed in isolated PSII preparations by using tandem mass spectrometry, which detects specific oxidative modifications of amino acid residues (Kale et al. 2017). In this case, the O_2_^**·**−^ that damages PSII is produced in PSII itself, most likely by reducing oxygen from Phe^−^ or Q_A_^−^ (Pospísil 2012; Kale et al. 2017). However, in the literature there is also a precedent for O_2_^**·**−^-mediated PSII photodamage by O_2_^**·**−^ which is produced at the acceptor side of PSI in the presence of methyl viologen in isolated thylakoids and intact leaves (Krieger-Liszkay et al. 2011).

Chloramphenicol is known to interact not only with PSI (Okada et al. 1991) but also with PSII (Rehman et al. 2016) by accepting electrons at the acceptor sides of both photosystems and then reducing oxygen and producing O_2_^**·**−^. Therefore, in principle either PSI, PSII, or both photosystems could be the site of superoxide production that damages PSII. Interestingly, the data presented in Figs. 1 and 2, as well as in Table 1 clearly show that the rate of PSII photodamage is increased practically to the same extent (ca. 37%) both in the WT and the PSI-less strain when chloramphenicol is used instead of lincomycin as protein synthesis inhibitor. This finding indicates that the O_2_^**·**−^ produced at PSI has a negligible contribution to the damage of PSII activity under our conditions, therefore the main source of O_2_^**·**−^ that damages PSII in the presence of chloramphenicol is PSII itself.

### PSII photodamage is enhanced in the absence of PSI

An important observation in our work is that light-induced loss of PSII activity in the presence of protein synthesis inhibitors proceeds faster in a PSI-less mutant than in the WT strain containing both PSI and PSII (Fig. 1 vs. Fig. 2, and Fig. 4). The net loss of PSII activity is determined by the balance of the competing photodamage and repair processes (Aro et al. 1993; Komenda et al. 2012; Järvi et al. 2015). Both sides of the balance can be affected by various factors. The rate of photodamage is linearly dependent on light intensity (Tyystjärvi and Aro 1996). However, it is also affected by the energetics of electron transfer in PSII. Namely, the increase of the free energy gap between Phe and Q_A_ provides protection against photodamage, whereas the decrease of the Phe and Q_A_ free energy gap enhances photodamage (Vass and Cser 2009; Rehman et al. 2013). This effect is related to the balance of radiative and non-radiative charge recombination pathways modulating the efficiency of singlet oxygen production, which is an important mediator of PSII photodamage (Cser and Vass 2007; Rehman et al. 2013). Modification of the environment of Q_A_ either by binding of herbicide molecules to the Q_B_ site or by site-directed mutagenesis also affects the rate of PSII photodamage. In both cases, the increase of E_m_(Q_A_/Q_A_^−^) provides protection against photodamage, whereas the decrease of E_m_(Q_A_/Q_A_^−^) enhances it (Krieger-Liszkay and Rutherford 1998; Fufezan et al. 2007).

The rate of PSII repair can also be influenced by several environmental factors. Salt, cold, moderate heat, oxidative stress and CO_2_ limitation can all inhibit PSII repair and therefore enhance net photodamage (Murata et al. 2007; Takahashi and Murata 2008). Production of reactive oxygen species, including singlet oxygen can also specifically inhibit the repair process by suppressing de novo synthesis of the D1 protein (Nishiyama et al. 2006). A large amount of ATP is also required for D1 protein synthesis, which is the key step of PSII repair, therefore the efficiency of cyclic electron flow, which contributes significantly to the pH gradient that drives ATP synthesis, can also influence the repair rate (Murata and Nishiyama 2018).

In the PSI-less *Synechocystis* mutant, the PSI electron transport pathways, which can oxidize the PQ pool are almost completely blocked. Therefore, the sustained reduction of the secondary quinone electron acceptors (PQ pool and Q_B_) maintains a highly reduced state of Q_A_, a condition also referred to as high excitation pressure (Maxwell et al. 1995). This condition enhances the production rate of singlet oxygen, which is one of the main mediators of PSII photodamage (Krieger-Liszkay et al. 2008; Vass and Cser 2009; Vass 2011; Fischer et al. 2013). Therefore, our data support the idea that enhancement of PSII photodamage in the absence of PSI is related to the lack of efficient electron transport beyond the PQ pool.

## Conclusions

Our data show that chloramphenicol at 200 μg mL^−1^, which is a frequently applied concentration in most photosynthesis related studies, enhances photodamage of PSII. Therefore, high chloramphenicol amounts should be avoided in studies, which aim at the determination of true rate of PSII photodamage. 100 μg mL^−1^ chloramphenicol appears to be a safe choice since it fully inhibits PSII repair without the side effect of enhanced photodamage rate. It is also of note that the side effect of chloramphenicol (and its recommended safe value) could be influenced by cell density of the culture and the temperature of the photoinhibitory treatment, which are expected to decrease and increase the enhancement of photodamage, respectively. Therefore, it is recommended to check the effect of the applied chloramphenicol concentration in comparison with lincomycin when new experimental conditions are designed. The chloramphenicol-induced enhancement of PSII photodamage is probably related to superoxide production. Therefore, superoxide should be considered as an important reactive oxygen species, besides singlet oxygen and hydroxyl radicals, that can damage PSII. This idea is in agreement with previous results showing superoxide-induced modification of the D1 reaction center protein (Kale et al. 2017) as well as superoxide production in PSII (Pospisil et al. 2019). The observed enhancement of PSII photodamage in the absence of PSI supports the important role of excitation pressure in influencing the rate of photodamage (Maxwell et al. 1995; Kornyeyev et al. 2010; Bersanini et al. 2014).

## Electronic supplementary material

Below is the link to the electronic supplementary material.

Supplementary file1 (DOCX 56 kb)

## Data Availability

All data presented are available in the form of figures, and tables in the main text and in the supplementary material.

## Abbreviations

Chl: Chlorophyll
DMBQ: 2,6-Dimethoxy-1,4-benzoquinone
PSI: Photosystem I
PSII: Photosystem II
