# Supplementary material for: Chloramphenicol enhances Photosystem II photodamage in intact cells of the cyanobacterium Synechocystis PCC 6803
Source: Photosynth Res. 2020 Sep 26;145(3):227–35. doi: 10.1007/s11120-020-00784-1 (PMC7541379; doi:10.1007/s11120-020-00784-1)
Supplement: Supplementary file 1 — Supplementary file1 (DOCX 56 kb) [file 11120_2020_784_MOESM1_ESM.docx]

Supplementary Material

**Chloramphenicol enhances Photosystem II photodamage in intact cells of the cyanobacterium *Synechocystis* PCC 6803**

Sandeesha Kodru^1,2^, Ateeq ur Rehman^1^ and Imre Vass^1,*^

^1^Institute of Plant Biology, Biological Research Centre, Szeged Hungary

^2^Doctoral School of Biology, University of Szeged

 *Corresponding author, 6726 Szeged, Temesvari krt. 62, Email: [vass.imre@brc.hu](mailto:vass.imre@brc.hu)

**Supplementary Figure 1. Effect of lincomycin and chloramphenicol concentration on PSII photodamage and recovery.** WT *Synechocystis* cultures were exposed to 500 μmol photons m^-2^s^-1^ intensity illumination (HL) followed by a recovery period at growth light intensity (LL:40 μmol photons m^-2^s^-1^). The cultures were either without addition of protein synthesis inhibitor (squares), or were treated with lincomycin (300 or 400 μg L^-1^, circles and stars, respectively), and with chloramphenicol (50, 100, and 200 μgL^-1^, shown by up triangles, down triangles, and lozenges, respectively). In case of 400 μgL^-1^ lincomycin only the photoinhibitory illumination was checked. PSII activity was assessed by measuring the rate of oxygen evolution in the presence of 0.5 mM DMBQ as an artificial acceptor. Data represent the mean of 4 to 6 biological replications with the indicated standard errors, and shown as percentage of the initial PSII activity, which was obtained from the growth-light-adapted cultures before the onset of high light illumination.

**Supplementary Table 1. A representative summary of chloramphenicol concentrations applied in photosynthesis related studies.**

| CAP conc.  (μg mL^-1^) | Experimental object | Reference |
| --- | --- | --- |
| 30 | Chlamydomonas reiinhardtii | Martin (1997) |
| 50 | Synechococcus 7942 | Komenda (1999) |
| 50 | Synechocystis 6803 | Komenda (1998) |
| 50 | Scenedesmus | Komenda (1998) |
| 65 | Spinach leaves | Demming-Adams (1993) |
|  |  |  |
| 100 | Spirulina | Vonshak (1994) |
| 100 | Bean leaves | Greer (1998) |
| 100 | Chlamydomonas reiinhardtii | Saradhi (2000) |
| 100 | Cosmarium | Stamenkovic (2013) |
|  |  |  |
| 200 | Barley | Stapel (1993) |
| 200 | Solanum tuberosum leaves | Havaux (1994) |
| 200 | Synechococcus 7942 | Gombos (1997) |
| 200 | Synechocystis 6803 | Nishiyama (2001) |
| 200 | Synechocystis 6803 | Nishiyama (2004) |
| 200 | Synechocystis 6803 | Nishiyama (2005) |
| 200 | Synechocystis 6803 | Takahashi (2005) |
| 200 | Synechocystis 6803 | Inoue (2011) |
| 200 | Synechococcus 7942 | Jimbo (2013) |
| 200 | Synechocystis 6803 | Kusama (2015) |
| 200 | Synechocystis 6803 | Ueno (2016) |
| 200 | Synechococcus elongatus | Sae-Tang (2016) |
| 250 | Chlorella | Vavilin (1995) |
|  |  |  |
| 300 | Wheat | Hurry (1992) |
| 323 | Ulva rotundata | Osmond (1993) |
| 323 | Coffea leaf | Da Matta (1997 |
| 300 | Soybean cell culture | Alfonso (2004) |
| 323 | Arabidopsis leaves | Takahashi (2010), |
| 300 | Rumex leaves | Zhang (2011) |
|  |  |  |
| 500 | Lemna gibba | Gong (1994) |
| 500 | Chlamydomonas reiinhardtii | Zhang (1997) |
| 500 | Chaetoceros gracilis | Nagao 2016 |
| 600 | Chlamydomonas reiinhardtii | Trebst (1996) |
| 600 | Chlamydomonas reiinhardtii | Depka (1998) |
| 600 | Chlamydomonas reiinhardtii | Jahns (2000) |
| 600 | Symbiodinium | Bhagooli (2013) |
|  |  |  |
| 1000 | Chlamydomonas reiinhardtii | Briantais (1988) |
| 1000 | Pumpkin leaves | Kettunen (1991) |
| 1000 | Pumpkin leaves | Tyystjarvi (1992 |
| 1000 | Ulva rotundata | Franklin (1992) |
| 1000 | Laminaria digitata | Rodriges (2002) |
| 1000 | Symbiodinium | Takahashi (2009) |
| 1000 | Symbiodinium | Takahashi (2013) |
| 1000 | Chlorella protothecoides | Zhang (2015) |
|  |  |  |
| 3000 | Pea leaves | Apostol (2001) |

**Supplementary Table 2. Light-induced decrease of PSII activity under different experimental conditions.** *Synechocystis* cultures were exposed to 500 μmol m^−2^s^−1^ light and their photosynthetic activity was followed as a function of illumination time by measuring the rate of oxygen evolution in the presence of DMBQ as artificial acceptor. The experiments were performed in photoautotrophically grown WT cells, without addition, and in the presence of protein synthesis inhibitors (300 and 400 μg mL^-1^ lincomycin, or 200 μg mL^-1^ chloramphenicol, abbreviated as CAP). The photoinhibitory treatments were also performed in a PSI-less *Synechocystis* mutant line, which cannot grow photoautotrophically, and therefore was cultured photomixotrophically in the presence of 5 mM glucose (PSI-less). As a control for the PSI-less mutant the WT cells were also grown photomixotrophically (WT-gluc). The PSI-less and WT-glucose cultures were exposed to the same light and inhibitor treatments as the photoautotrophically grown WT. The mean values and standard deviations were calculated from 4-6 biological replicates. ANOVA analysis of the data was performed by using the OriginPro 2018 software, with the Tukey Test option for means comparison. Bold values in the ANOVA table indicate the probability values, which are less than the 0.05 threshold set for a significant difference between the two corresponding mean values shown in the 1^st^ column.

**References**

Alfonso M, Collados R, Yruela I, Picorel R (2004) Photoinhibition and recovery in a herbicide-resistant mutant from Glycine max (L.) Merr. cell cultures deficient in fatty acid unsaturation. Planta 219 (3):428-439. doi:10.1007/s00425-004-1242-5

Apostol S, Briantais JM, Moise N, Cerovic ZG, Moya I (2001) Photoinactivation of the photosynthetic electron transport chain by accumulation of over-saturating light pulses given to dark adapted pea leaves. Photosynthesis Research 67 (3):215-227. doi:10.1023/a:1010676618028

Bhagooli R (2013) Inhibition of Calvin-Benson cycle suppresses the repair of photosystem II in *Symbiodinium*: implications for coral bleaching. Hydrobiologia 714:183-190

Briantais JM, Cornic G, Hodges M (1988) The modification of chlorophyll fluorescence of Chlamydomonas reinhardtii by photoinhibition and chloramphenicol addition suggests a form of Photosystem II less susceptible to degradation. FEBS Lett 236:226-230

Chen Z, Lu GF, Chen S, Chen XW (2011) Light dependency of photosynthetic recovery during wetting and the acclimation of photosynthetic apparatus to light fluctuation in a terrestrial cyanobacterium Nostoc commune. Journal of Phycology 47 (5):1063-1071. doi:10.1111/j.1529-8817.2011.01033.x

Da Matta FM, Maestri M (1997) Photoinhibition and recovery of photosynthesis in Coffea arabica and C-canephora. Photosynthetica 34 (3):439-446. doi:10.1023/a:1006824404141

Demming-Adams B, Adams WWI (1993) The xanthophyll cycle, protein turnover, and the high light tolerance of sun-acclimated leaves. Plant Physiol 103:1413-1420

Depka B, Jahns P, Trebst A (1998) b-carotene to zeaxanthin conversion in the rapid turnover of the D1 protein of Photosystem II. FEBS Lett 424:267-270

Franklin LA, Levavasseur G, Osmond CB, Henley WJ, Ramus J (1992) 2 components of onset and recovery during photoinhibition of Ulva-rotundata. Planta 186 (3):399-408

Gombos Z, Kanervo E, Tsvetkova N, Sakamoto T, Aro EM, Murata N (1997) Genetic enhancement of the ability to tolerate photoinhibition by introduction of unsaturated bonds into membrane glycerolipids. Plant Physiol 115:551-559

Gong H (1994) Light-dependent degradation of the Photosystem II D1 protein is retarded by inhibitors of chloroplast transcription and translation: possible involvement of a chloroplast-encoded proteinase. Biochim Biophys Acta 1188:422-426

Greer DH (1998) Photoinhibition of photosynthesis in dwarf bean (Phaseolus vulgaris L.) leaves: Effect of sink-limitations induced by changes in daily photon receipt. Planta 205 (2):189-196. doi:10.1007/s004250050311

Havaux M (1994) Temperature-dependent modulation of the photoinhibition-sensitivity of Photosystem-II in Solanum tuberosum leaves. Plant Cell Physiol 35 (5):757-766. doi:10.1093/oxfordjournals.pcp.a078654

Hurry VM, Huner NPA (1992) Effect of cold hardening on sensitivity of winter and spring wheat leaves to short-term photoinhibition and recovery of photosynthesis. Plant Physiology 100 (3):1283-1290. doi:10.1104/pp.100.3.1283

Inoue S, Ejima K, Iwai E, Hayashi H, Appel J, Tyystjärvi E, Murata N, Nishiyama Y (2011) Protection by a-tocopherol of the repair of photosystem II during photoinhibition in *Synechocystis* sp. PCC 6803. Biochim Biophys Acta 1807:236-241

Jahns P, Depka B, Trebst A (2000) Xanthophyll cycle mutants from Chlamydomonas reinhardtii indicate a role for zeaxanthin in the D1 protein turnover. Plant Physiol Biochem 38 (5):371-376. doi:10.1016/s0981-9428(00)00753-1

Jimbo H, Noda A, Hayashi H, Nagano T, Yumoto I, Orikasa Y, Okuyama H, Nishiyama Y (2013) Expression of a highly active catalase VktA in the cyanobacterium *Synechococcus elongatus* PCC 7942 alleviates the photoinhibition of photosystem II. Photosynth Res 117 (1-3):509-515

Kettunen R, Tyystjarvi E, Aro EM (1991) D1 protein degradation during photoinhibition of intact leaves. A modification of the D1 protein precedes degradation. FEBS Lett 290:153-156

Komenda J (1998) Photosystem 2 photoinactivation and repair in the Scenedesmus cells treated with herbicides DCMU and BNT and exposed to high irradiance. Photosynthetica 35 (3):477-480. doi:10.1023/a:1006984906965

Komenda J, Koblizek M, Masojidek J (1999) The regulatory role of photosystem II photoinactivation and de novo protein synthesis in the degradation and exchange of two forms of the D1 protein in the cyanobacterium Synechococcus PCC 7942. J Photochem Photobiol B-Biol 48 (2-3):114-119. doi:10.1016/s1011-1344(99)00025-1

Komenda J, Masojidek J (1998) The effect of Photosystem II inhibitors DCMU and BNT on the high-light induced D1 turnover in two cyanobacterial strains Synechocystis PCC 6803 and Synechococcus PCC 7942. Photosynthesis Research 57 (2):193-202. doi:10.1023/a:1006015214868

Kusama Y, Inoue S, Jimbo H, Takaichi S, Sonoike K, Hihara Y, Nishiyama Y (2015) Zeaxanthin and echinenone protect the repair of Photosystem II from inhibition by singlet oxygen in *Synechocystis* sp. PCC 6803. Plant Cell Physiol 0 (0):1-11 doi:10.1093/pcp/pcv1018

Martin RE, Thomas DJ, Tucker DE, Herbert SK (1997) The effects of photooxidative stress on photosystem I measured in vivo in Chlamydomonas. Plant Cell and Environment 20 (12):1451-1461. doi:10.1046/j.1365-3040.1997.d01-47.x

Nagao R, Tomo T, Narikawa R, Enami I, Ikeuchi M (2016) Conversion of photosystem II dimer to monomers during photoinhibition is tightly coupled with decrease in oxygen-evolving activity in the diatom Chaetoceros gracilis. Photosynthesis Research 130 (1-3):83-91. doi:10.1007/s11120-016-0226-1

Nishiyama Y, Allakhverdiev SI, Yamamoto H, Hayashi H (2004) Singlet oxygen inhibits the repair of Photosystem II by suppressing the translation elongation of the D1 protein in *Synechocystis* sp. PCC 6803. Biochemistry 43:11321-11330

Osmond CB, Ramus J, Levavasseur G, Franklin LA, Henley WJ (1993) Fluorescence quenching during photosynthesis and photoinhibition of Ulva-rotundta blid. Planta 190 (1):97-106

Rodrigues MA, dos Santos CP, Young AJ, Strbac D, Hall DO (2002) A smaller and impaired xanthophyll cycle makes the deep sea macroalgae Laminaria abyssalis (Phaeophyceae) highly sensitive to daylight when compared with shallow water Laminaria digitata. Journal of Phycology 38 (5):939-947. doi:10.1046/j.1529-8817.2002.t01-1-01231.x

Sae-Tang P, Hihara Y, Yumoto I, Orikasa Y, Okuyama H, Nishiyama Y (2016) Overexpressed Superoxide Dismutase and Catalase Act Synergistically to Protect the Repair of PSII during Photoinhibition in Synechococcus elongatus PCC 7942. Plant Cell Physiol 57 (9):1899-1907. doi:10.1093/pcp/pcw110

Saradhi PP, Suzuki I, Katoh A, Sakamoto A, Sharmila P, Shi D-J, Murata N (2000) Protection against the photo-induced inactivation of the Photosystem II complex by abscisic acid. Plant Cell Environ 23:711-718

Stamenkovic M, Hanelt D (2013) Protection Strategies of Cosmarium strains (Zygnematophyceae, Streptophyta) Isolated from Various Geographic Regions Against Excessive Photosynthetically Active Radiation. Photochemistry and Photobiology 89 (4):900-910. doi:10.1111/php.12083

Stapel D, Kruse E, Kloppstech K (1993) The protective effect of heat-shock proteins against photoinhibition under heat-shock in barley (Hordeum-vulgare). J Photochem Photobiol B-Biol 21 (2-3):211-218. doi:10.1016/1011-1344(93)80185-c

Takahashi S, Milward SE, Yamori W, Evans JR, Hillier W, Badger MR (2010) The solar action spectrum of Photosystem II damage. Plant Physiol 153:988-993

Takahashi S, Yoshioka-Nishimura M, Nanba D, Badger MR (2013) Thermal acclimation of the symbiotic alga *Symbiodinium* spp. alleviates photobleaching under heat stress. Plant Physiol 161:477-485

Trebst A, Soll-Bracht E (1996) Cycloheximide retards high light driven D1 protein degradation in *Chlamydomonas reinhardtii*. Plant Sci 115:191-197

Tyystjarvi E, Aki-Yrkkö K, Kettunen R, Aro E-M (1992) Slow degradation of the D1 protein is related to the susceptibility of lowülightügrown pumpkin plants to photoinhibition. Plant Physiol 100:1310-1317

Ueno M, Sae-Tang P, Kusama Y, Hihara Y, Matsuda M, Hasunuma T, Nishiyama Y (2016) Moderate Heat Stress Stimulates Repair of Photosystem II During Photoinhibition in Synechocystis sp PCC 6803. Plant Cell Physiol 57 (11):2417-2426. doi:10.1093/pcp/pcw153

Vavilin DV, Polynov VA, Matorin DN, Venediktov PS (1995) Sublethal concentrations of copper stimulate Photosystem-II photoinhibition in Chlorella-pyrenoidosa.. J Plant Physiol 146 (5-6):609-614. doi:10.1016/s0176-1617(11)81922-x

Vonshak A, Torzillo G, Tomaseli L (1994) USE OF CHLOROPHYLL FLUORESCENCE TO ESTIMATE THE EFFECT OF PHOTOINHIBITION IN OUTDOOR CULTURES OF SPIRULINA-PLATENSIS. Journal of Applied Phycology 6 (1):31-34. doi:10.1007/bf02185901

Zhang LP, Niyogi KK, Baroli I, Nemson JA, Grossman AR, Melis A (1997) DNA insertional mutagenesis for the elucidation of a Photosystem II repair process in the green alga Chlamydomonas reinhardtii. Photosynthesis Research 53 (2-3):173-184. doi:10.1023/a:1005867709441

Zhang LT, He ML, Liu JG, Li L (2015) Role of the mitochondrial alternative oxidase pathway in hydrogen photoproduction in Chlorella protothecoides. Planta 241 (4):1005-1014. doi:10.1007/s00425-014-2231-y

Zhang LT, Zhang ZS, Gao HY, Xue ZC, Yang C, Meng XL, Meng QW (2011) Mitochondrial alternative oxidase pathway protects plants against photoinhibition by alleviating inhibition of the repair of photodamaged PSII through preventing formation of reactive oxygen species in Rumex K-1 leaves. Physiologia Plantarum 143 (4):396-407. doi:10.1111/j.1399-3054.2011.01514.x
